# Association between Statin Use and Sepsis Risk in Patients with Dementia: A Retrospective Cohort Study

**DOI:** 10.3390/ijerph16091626

**Published:** 2019-05-09

**Authors:** Liang-Tsai Yeh, Chuan-Yi Tang, Shun-Fa Yang, Han-Wei Yeh, Ying-Tung Yeh, Yu-Hsun Wang, Ming-Chih Chou, Chao-Bin Yeh, Chi-Ho Chan

**Affiliations:** 1 Institute of Medicine, Chung Shan Medical University, Taichung 402, Taiwan; 68990@cch.org.tw (L.-T.Y.); ysf@csmu.edu.tw (S.-F.Y.); ycb@csmu.edu.tw (M.-C.C.); 2 Department of Anesthesiology, Changhua Christian Hospital, Changhua 500, Taiwan; 3 Department of Computer Science and Information Engineering, Providence University, Taichung City 433, Taiwan; cytang@pu.edu.tw; 4 Department of Medical Research, Chung Shan Medical University Hospital, Taichung 402, Taiwan; cshe731@csh.org.tw; 5 School of Medicine, Chang Gung University, Taoyuan City 333, Taiwan; george66889@gmail.com; 6 Graduate School of Dentistry, Chung Shan Medical University, Taichung 402, Taiwan; yehtungtung@hotmail.com; 7 School of Dentistry, Chung Shan Medical University, Taichung 402, Taiwan; 8 Department of Dentistry, Chung Shan Medical University Hospital, Taichung 402, Taiwan; 9 Department of Emergency Medicine, School of Medicine, Chung Shan Medical University, Taichung 402, Taiwan; 10 Department of Emergency Medicine, Chung Shan Medical University Hospital, Taichung 402, Taiwan; 11 Department of Microbiology and Immunology, Chung Shan Medical University, Taichung 402, Taiwan

**Keywords:** statin, sepsis, retrospective cohort study

## Abstract

This study investigated the association of statin use with sepsis risk in patients with dementia. This retrospective cohort study was conducted in Taiwan by using data from the National Health Insurance Research Database. We identified and enrolled 308 patients with newly diagnosed dementia who used statin after dementia diagnosis. These patients were individually propensity score matched (1:1) according to age, sex, hypertension, hyperlipidemia, diabetes, cerebrovascular disease, renal disease, liver disease, asthma, malignancy, parkinsonism, and dementia drugs used (donepezil, rivastigmine, galantamine, and memantine) with 251 controls (statin non-users). A Cox proportional hazard model was used to estimate the adjusted hazard ratio for sepsis in statin users and non-users. After adjustment for other confounding factors, the incidence of sepsis in statin users was 1.42-fold higher than that in non-users (95% confidence interval = 0.81–2.5). In conclusion, our analysis showed no positive association of sepsis with statin use in patients with dementia.

## 1. Introduction

Statins are a group of drugs that are widely used to decrease blood cholesterol through the inhibition of hydroxymethylglutaryl-CoA reductase enzyme, which is a critical enzyme for cholesterol production in mevalonate metabolism. According to their synthetic nature and molecular structure, statins can be divided into closed-ring pro-drugs (i.e., lovastatin and simvastatin) and synthetic compounds (i.e., fluvastatin, atorvastatin, rosuvastatin, and pitavastatin) [1]. Additionally, regarding their clinical use, statins have pleiotropic functions. For instance, statins can be used for the prevention of primary and secondary coronary heart disease (CHD), as hypercholesterolemia is a major risk factor for CHD [2]. Furthermore, statins have several benefits in treating patients with underlying diseases such as contrast-induced nephropathy, chronic obstructive pulmonary disease, and community-acquired pneumonia [3,4,5].

However, statins have several adverse effects such as myalgia, cramps, and skeletal muscle weakness as well as myopathy and rhabdomyolysis in some cases [6,7]. Moreover, statin treatment might cause organ damage and increase the risk of several diseases. For example, statins may lead to hepatotoxicity, increased diabetes risk, and cataract development [8,9,10,11]. The effect of statins on patients with neurological disorder and dementia has been broadly discussed in the past few years. Owing to the antioxidant, anti-inflammatory, and antiplatelet effects, the therapeutic potential of statin in neurological disorders such as Alzheimer’s disease (AD), Parkinson’s disease (PD), multiple sclerosis, and brain tumors has been discussed [12]. A cohort study indicated that long-term statin use decreased the risk of AD and dementia in elderly African Americans [13]. A review study of the effects of statins on neurodegenerative diseases provided controversial results. Although evidence has indicated that statins may play a protective role in neurodegenerative diseases such as dementia (i.e., AD and PD), many studies have reported no association between statin use and decelerated memory loss and cognitive function in patients with AD and PD [14]. Risk factors for statin adverse effects have been revised extensively. They included dose subscription of statins, drug–drug interaction, age, gender, and comorbidities (renal insufficiency, hepatic dysfunction, alcohol abuse, hypertension, diabetes mellitus, and obesity, etc.). Other risk factors might be related to genetic mutations of mitochondrial dysfunction and genetic variants. For instance, a study showed that more than 60% of myopathy cases were correlated with the C-allele of the rs4149056 single-nucleotide polymorphism on chromosome 12 [15]. In terms of the muscle adverse effect, the mechanism involved the mitochondrial impairment. This could also be reflected by the supplementation of Q10 co-enzyme, which was involved in muscle metabolism, to reduce the muscle adverse effects [16]. According to an updated review of the mechanisms associated with the adverse effects of statins, statins induced reactive oxygen species and oxidative stress that promoted organ damage [17].

Sepsis involves progressive organ dysfunction and is a major cause of death through infection. The seriousness of sepsis is determined on the basis of pathogens and several factors such as race, age, gender, and comorbidities [18]. Owing to its complicated diagnosis, the definition of sepsis has been revised continuously. To reduce the mortality of patients with sepsis, an early diagnosis and optimized treatment are necessary. According to the Third International Consensus Definitions for Sepsis and Septic Shock (Sepsis-3), sepsis has been redefined as evidence of infection plus life-threatening organ dysfunction clinically characterized by an acute change of 2 points or greater in the Sequential Organ Failure Assessment score [19,20].

According to Alzheimer Disease International, the total number of patients with dementia in the world is approximately 50 million. This number is estimated to reach 131.5 million in 2050. In 2017, there were approximately 150,000 people in Taiwan aged ≥60 years [21]. Statins are the most popular hypolipidemic drugs in clinical use. These drugs might protect patients from dementia risk. In addition, statins may also decrease sepsis risk in patients with different comorbidities [22,23,24,25]. It is rational to hypothesize that statins can also decrease the risk of developing sepsis in dementia patients. Thus, the aim of this study was to analyze whether a positive relationship exists between statin use and sepsis development in patients with dementia.

## 2. Materials and Methods

### 2.1. Data Source

The Taiwan National Health Insurance Research Database contains the claims data of >99% of Taiwan’s entire population of 23 million. The Longitudinal Health Insurance Database (LHID) contains the data of one million randomly sampled patients from the National Health Insurance Research Database. It includes information on medical expenditures, disease diagnosis, prescriptions, medical operation, and procedures. The identification numbers of all patients were encrypted. The study was approved by the Ethical Review Board of Chung Shan Medical University Hospital (CSMU No. CS18096).

### 2.2. Study Groups

This study used a retrospective cohort study design. We selected patients aged ≥40 years who had newly diagnosed dementia (International Classification of Diseases, Ninth Revision, Clinical Modification (ICD-9-CM) codes 290.0–290.4, 294.1, 331.0–331.2, and 331.82) from 2010 to 2012. To confirm the accuracy of the diagnosis, we only included patients who were diagnosed by a neurologist or psychiatrist and underwent examination with Mini-Mental Status Examination, brain computed tomography, or brain magnetic resonance imaging. The index date was defined as the first date of statin use after dementia diagnosis. Seven statins were included in this study: atorvastatin, fluvastatin, lovastatin, pitavastatin, pravastatin, rosuvastatin, and simvastatin. The non-statin group included those who had never used statin from 2009 to 2013.

### 2.3. Main Outcome Measurement

The main outcome was sepsis diagnosis (ICD-9-CM codes 038 and 995.91) after the index date. The study end point was sepsis occurrence, 31 December 2013, or withdrawal from the national health insurance program, whichever occurred first. To confirm new-onset sepsis, we excluded those who were diagnosed with sepsis before the index date.

### 2.4. Covariates and Matching

Baseline characteristics were age, gender, hypertension (ICD-9-CM codes 401–405), hyperlipidemia (ICD-9-CM codes 272.0–272.4), diabetes (ICD-9-CM code 250), cerebrovascular disease (ICD-9-CM codes 430–438), renal disease (ICD-9-CM codes 582, 583.0–583.7, 585, 586, and 588), liver disease (ICD-9-CM codes 456.0–456.21, 571.2, 571.4, 571.5, 571.6, and 572.2–572.8), asthma (ICD-9-CM codes 493), malignancy (ICD-9-CM codes 140–172.9, 174–195.8, and 200–208.9), and parkinsonism (ICD-9-CM code 332). This study included comorbidities diagnosed during at least two outpatient visits or once during admission 1 year before the index date. Antidementia medication use (donepezil, rivastigmine, galantamine, and memantine) was defined as the ever use of antidementia medication during the study period.

First, 1:10 matching by age (±5 years), gender, and dementia date (±180 days) was used to provide an index date for the non-statin group that corresponded to that of the statin group in order to have the same starting point for both groups. After matching, we excluded those with sepsis diagnosis before the index date. To balance the heterogeneity between the two groups, propensity score matching (1:1) was performed for the non-statin group according to age, gender, comorbidities, and antidementia medication use. The propensity score was estimated through logistic regression. Statin and non-statin use were binary outcomes. By matching the propensity score, we could determine similarities among both groups.

### 2.5. Statistical Analysis

The chi-square test and independent *t* test were used to compare the statin and non-statin groups, as appropriate. Kaplan–Meier analysis was used to draw the plot of the cumulative incidence of sepsis between statin group and non-statin group. A log-rank test was used to estimate the significance. A Cox proportional hazard model was used to estimate the hazard ratios of sepsis between the two groups. The analytical statistical software used was SPSS V.18.0 (SPSS, Chicago, IL, USA). Significance was defined as *p* < 0.05.

## 3. Results

**Figure 1:**
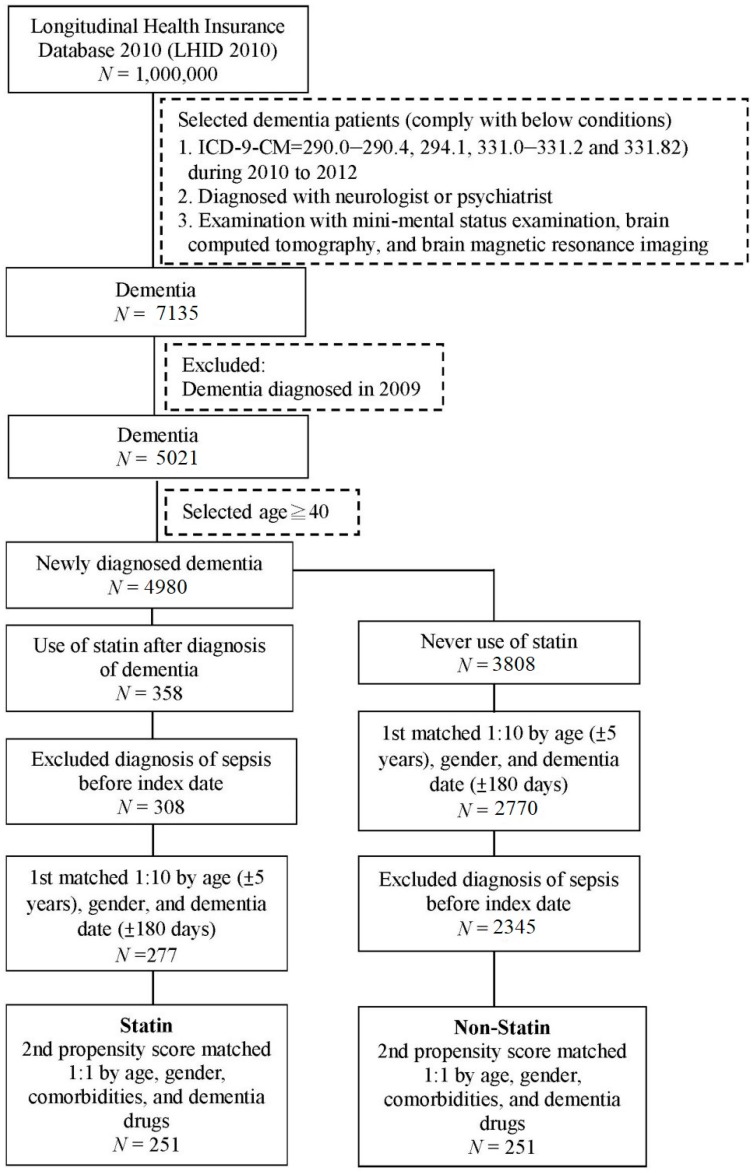
Flow chart for patient selection.

In this study, we recruited 4980 patients aged ≥40 who had newly diagnosed dementia from LHID 2010 (Figure 1). These patients were divided into statin (*n* = 358) and non-statin (*n* = 3808) groups. After excluding patients with sepsis diagnosis before the index date, 308 patients were included in the statin group. Next, we performed 1:10 matching by age, sex, and dementia date to determine an index date for the non-statin group corresponding to that of the statin group. Finally, 251 statin users and an equal number of non-users were further screened through propensity score matching by age, gender, comorbidities, and antidementia medication use (Figure 1). Both groups (statin and non-statin) were similar in terms of age, sex, comorbidities, comorbidity severity, and drug use. For instance, the percentage of male patients in the statin and non-statin groups was 45.8% and 44.2%, respectively (Table 1).

**Figure 2:**
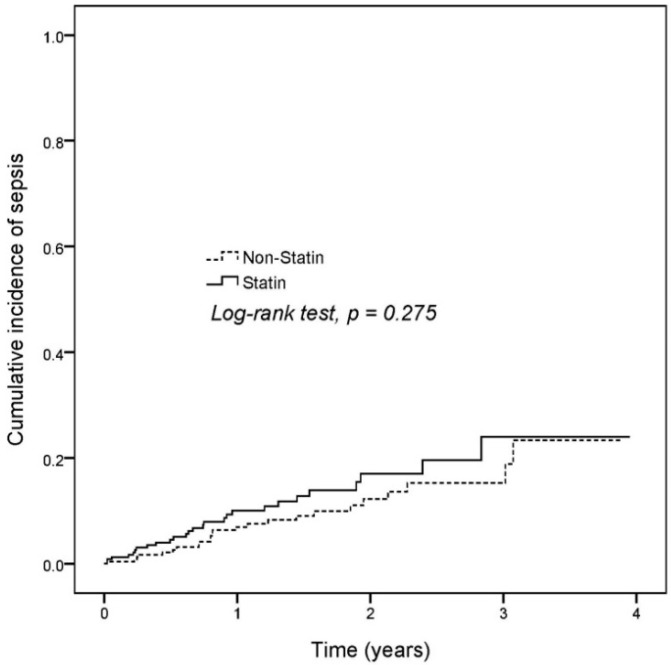
Kaplan–Meier curves of the cumulative incidence of sepsis in dementia patients who did and did not use statin.

We estimated the cumulative incidence of sepsis in statin users and non-users. The Kaplan–Meier curve depicted an increasing incidence of sepsis in statin users compared with that in non-users for the first 3 years, but both groups showed similar sepsis incidence in the fourth year (log-rank test, *p* = 0.2757; Figure 2). According to the Cox proportional hazard model, statin users had a 1.42-fold higher risk of sepsis than non-users. Furthermore, age, male, diabetes, cerebrovascular disease, renal disease, liver disease, asthma, and parkinsonism were associated with a positive sepsis risk (Table 2). A subgroup analysis was performed between the statin and non-statin groups. Male patients in the statin group had a higher risk of sepsis (hazard ratio = 2.29, 95% confidence interval = 1.01–5.21), but the *p* value for interaction was not statistically significant by gender (Table 3).

## 4. Discussion

Statins have been widely used for the treatment of hypercholesterolemia. As statins reduce cholesterol precipitation on the wall of blood vessels, they have been administrated for preventing coronary artery diseases and even stroke [1,5]. In previous studies, the benefits and adverse effects of statins have been discussed. Despite their therapeutic values, statins have certain adverse effects. For example, when patients with active Graves′ orbitopathy were treated with intravenous glucocorticoids and statins (i.e., simvastatin or rosuvastatin) simultaneously, liver dysfunction occurred. Liver function recovered after statins were discontinued [26]. The possible causes of hepatotoxicity due to statins might include genetic polymorphism of the metabolic enzyme, drug–drug interaction, and prolonged usage [10]. Furthermore, statins may damage the skeletal muscle. A study named “Statins on Muscle Performance” indicated that prolonged statin use increase creatine kinase production in skeletal muscle, resulting in muscle injury and myalgia [6]. High-intensity treatment using statins increases the risk of diabetes, and lipophilic statins (i.e., rosuvastatin and pravastatin) are more diabetogenic [8,9].

The healthy user effect has been a form of research bias present in some previous studies on statin use. In observational research studies, those who take statins tend to be healthier than those who do not, and therefore are more likely to experience favorable outcomes [27]. With this in mind, our research group enrolled subjects with dementia and matched them by propensity score. As such, the heterogeneity of the two groups would be reduced. One large observational research study suggested that use of statin would reduce the mortality associated with infection [22]. Another large randomized placebo-controlled trial indicated that use of statin was not associated with risk of infection [28]. The difference between the two studies was the process of random allocation. To reduce the potential influence of the healthy user effect, the result would lead to a non-significant effect. Moreover, the association of statin use with sepsis outcomes in patients with particular diseases has been widely studied for many years, and controversy exists between observational studies and randomized trial studies. The majority of observational studies have indicated that pretreatment with statins before being admitted to the hospital results in improved sepsis outcomes. For instance, a study that investigated sepsis risk in intensive care unit patients found a reduced percentage of sepsis development among patients with prior treatment with statins [24]. In another population-based cohort study, atherosclerotic patients who received statins had a subsequently lower risk of sepsis [29]. A systemic review of 22 studies on statins and infection was conducted. The majority of the studies suggested that statins might have a positive role in the treatment of patients with sepsis and infection [23]. Recently, a cohort study in Taiwan showed that preadmission statin therapy before sepsis development was associated with a 12% reduction in mortality when compared with patients who never received statin. However, the effect was obvious only for patients with less serious conditions [30]. In contrast to observational studies, meta-analyses of randomized controlled trials addressing statins and infection failed to show a beneficial effect of statins on infection-related outcomes [27,28]. The different findings might be the result of several relevant factors including indication bias, healthy tolerator effects, and healthy user effects, which are pivotal for preventive drugs.

Furthermore, animal studies have suggested that statin therapy may improve certain organs damaged through sepsis. Statin therapy with atorvastatin, pravastatin, or simvastatin, but not with fluvastatin, in septic mice prolonged their survival time [25]. By using elderly mice as an animal model, simvastatin was shown to improve sepsis-induced acute kidney injury through direct effects on the renal vasculature and reversal of tubular hypoxia, and it had a systemic anti-inflammatory effect [31]. In a rat model, simvastatin had a protective effect on myocardial depression caused by sepsis [32]. The effect may be mediated through the inhibition of the production of proinflammatory and inflammatory cytokines such as tumor necrosis factor-alpha, interleukin (IL)-1 beta, IL-6, Monocyte Chemoattractant Protein-1 (MCP-1), and NO [32]. These results indicate that statins may ameliorate organ damage caused by sepsis.

Moreover, a randomized trial of the effect of atorvastatin in patients with severe sepsis indicated that prior statin users had lower 28-day mortality than placebo users [33]. However, a meta-analysis of nine randomized trial studies concluded that statin therapy had no effect on mortality outcomes in patients with sepsis compared with placebo users [34]. In a previous double-blind investigation, treatment of hypercholesterolemia with lovastatin did not cause psychological distress or substantially alter cognitive function [35]. A similar study was extended to simvastatin. They found only minor decrements in cognitive functioning with the statin [36]. In our study, we observed that statin use had no direct association with sepsis risk in patients with dementia.

Additionally, many diseases are risk factors for sepsis. In our study, dementia patients with underlying diseases, such as diabetes, cerebrovascular diseases, renal diseases, liver diseases, asthma, and PD, had a higher risk of sepsis. This result corresponds with those of previous studies [37,38,39,40]. However, our study still has certain limitations. First, there are at least six types of statins. Different statins might have different effects on patients with dementia. Second, patients’ physiological data, laboratory data, and personal behavioral information (such as drinking and smoking habits and body mass index) that might affect sepsis occurrence are not available in the datasets. Third, patients’ medication compliance could not be determined in this study. The protective effect of statins in the development of sepsis in patients with dementia may be underestimated.

## 5. Conclusions

In conclusion, our study showed no positive association between statin use and sepsis development in patients with dementia. Moreover, patients with underlying diseases, such as diabetes, cerebrovascular disease, renal disease, liver disease, asthma, and PD, had a higher risk of sepsis. However, we found that the incidence of sepsis tended to be higher in statin users than in non-users. Hence, physicians should exercise caution when prescribing statins to patients.

## Figures and Tables

**Table 1:** Demographic characteristics of statin and non-statin users.

Variable	Statin (*N* = 251)		Non-Statin (*N* = 251)		
	*n*	%	*n*	%	*p*-Value
Age, mean ± SD	77.8 ± 8.8		77.5 ± 7.8		0.715
Sex					0.720
Female	136	54.2	140	55.8	
Male	115	45.8	111	44.2	
Hypertension	208	82.9	216	86.1	0.324
Hyperlipidemia	131	52.2	119	47.4	0.284
Diabetes	130	51.8	146	58.2	0.151
Cerebrovascular disease	155	61.8	151	60.2	0.714
Renal disease	38	15.1	45	17.9	0.400
Liver disease	9	3.6	10	4.0	0.815
Asthma	17	6.8	15	6.0	0.715
Malignancy	21	8.4	21	8.4	1.000
Parkinsonism	30	12.0	32	12.7	0.786
Dementia drugs	18	7.2	21	8.4	0.617

**Table 2:** Cox proportional hazards model of sepsis and exposure to statin.

Variable	*N*	No. of Sepsis Event	Crude Hazard Ratio (HR)	95% CI	Adjusted Hazard Ratio (HR) ^†^	95% CI
Statin						
No	251	24	1		1	
Yes	251	27	1.36	0.78–2.36	1.42	0.81–2.50
Age	502	51	1.03	0.99–1.07	1.03	0.99–1.07
Sex						
Female	276	26	1		1	
Male	226	25	1.25	0.72–2.17	1.16	0.66–2.05
Hypertension	424	43	0.93	0.44–1.99	0.92	0.43–2.00
Hyperlipidemia	250	21	0.68	0.39–1.19	0.76	0.43–1.36
Diabetes	276	29	1.15	0.66–1.99	1.35	0.75–2.45
Cerebrovascular disease	306	37	1.65	0.89–3.05	1.66	0.87–3.17
Renal disease	83	10	1.40	0.7–2.8	1.43	0.70–2.92
Liver disease	19	4	2.11	0.76–5.86	2.09	0.71–6.14
Asthma	32	7	2.03	0.91–4.53	2.02	0.90–4.55
Malignancy	42	3	0.73	0.23–2.33	0.78	0.24–2.56
Parkinsonism	62	12	2.12	1.11–4.05	1.93	0.99–3.79
Dementia drugs	39	3	0.66	0.21–2.12	0.84	0.25–2.80

† Adjusted for statin, age, sex, hypertension, hyperlipidemia, diabetes, cerebrovascular disease, renal disease, liver disease, asthma, malignancy, parkinsonism, and dementia drugs.

**Table 3:** Subgroup analysis of Cox proportional hazards model of sepsis.

Variable	Statin		Non-Statin			
	*N*	No. of Sepsis Event	*N*	No. of Sepsis Event	HR	95% CI
Age						
<70	49	4	33	1	2.78	0.31–24.84
≥70	202	23	218	23	1.42	0.79–2.53
*p* for interaction = 0.544						
Gender						
Female	136	11	140	15	0.86	0.40–1.88
Male	115	16	111	9	2.29 *	1.01–5.21
*p* for interaction = 0.124						

* *p* < 0.05.
